# Massachusetts Alumni Association of McGill Veterinary Department

**Published:** 1898-07

**Authors:** 


### Massachusetts Alumni Association of McGill Veterinary Department.

Officers: President, Dr. J. R. McLaughlin, of Newton; Secretary, Dr. John M. Parker, of Haverhill. This association was organized about four years ago, and has annually reëlected the same officers. The annual reunion and banquet is held in February of each year, and informal meetings in June and November. The association has twice been the invited guest of the Massachusetts Veterinary Association on their June outing down the bay, which has added much to the pleasure of the organization.

A strong effort will be put forth to hold a grand reunion of the graduates of old Montreal, now the Veterinary Department of McGill, in February, 1899, at the old college buildings in Union Avenue, Montreal.

Montreal graduates are scattered from the Sandwich Islands, California, Maine, every province in Canada, and most of the States of the Union, but it is earnestly hoped that every class will have a number of representatives in February next.

